# Statistical Machines for Trauma Hospital Outcomes Research: Application to the PRospective, Observational, Multi-Center Major Trauma Transfusion (PROMMTT) Study

**DOI:** 10.1371/journal.pone.0136438

**Published:** 2015-08-21

**Authors:** Sara E. Moore, Anna Decker, Alan Hubbard, Rachael A. Callcut, Erin E. Fox, Deborah J. del Junco, John B. Holcomb, Mohammad H. Rahbar, Charles E. Wade, Martin A. Schreiber, Louis H. Alarcon, Karen J. Brasel, Eileen M. Bulger, Bryan A. Cotton, Peter Muskat, John G. Myers, Herb A. Phelan, Mitchell J. Cohen, PROMMTT Study Group

**Affiliations:** 1 Division of Biostatistics, University of California, Berkeley, California, United States of America; 2 Division of General Surgery, Department of Surgery, School of Medicine, University of California San Francisco, San Francisco, California, United States of America; 3 Center for Translational Injury Research, Division of Acute Care Surgery, Department of Surgery, Medical School, University of Texas Health Science Center at Houston, Houston, Texas, United States of America; 4 Division of Clinical and Translational Sciences, Department of Internal Medicine, Medical School, University of Texas Health Science Center at Houston, Houston, Texas, United States of America; 5 Division of Trauma, Critical Care and Acute Care Surgery, School of Medicine, Oregon Health & Science University, Portland, Oregon, United States of America; 6 Division of Trauma and General Surgery, Department of Surgery, School of Medicine, University of Pittsburgh, Pittsburgh, Pennsylvania, United States of America; 7 Division of Trauma and Critical Care, Department of Surgery, Medical College of Wisconsin, Milwaukee, Wisconsin, United States of America; 8 Division of Trauma and Critical Care, Department of Surgery, School of Medicine, University of Washington, Seattle, Washington, United States of America; 9 Division of Trauma/Critical Care, Department of Surgery, College of Medicine, University of Cincinnati, Cincinnati, Ohio, United States of America; 10 Division of Trauma, Department of Surgery, School of Medicine, University of Texas Health Science Center at San Antonio, San Antonio, Texas, United States of America; 11 Division of Burn/Trauma/Critical Care, Department of Surgery, Medical School, University of Texas Southwestern Medical Center at Dallas, Dallas, Texas, United States of America; University of Pennsylvania, UNITED STATES

## Abstract

Improving the treatment of trauma, a leading cause of death worldwide, is of great clinical and public health interest. This analysis introduces flexible statistical methods for estimating center-level effects on individual outcomes in the context of highly variable patient populations, such as those of the PRospective, Observational, Multi-center Major Trauma Transfusion study. Ten US level I trauma centers enrolled a total of 1,245 trauma patients who survived at least 30 minutes after admission and received at least one unit of red blood cells. Outcomes included death, multiple organ failure, substantial bleeding, and transfusion of blood products. The centers involved were classified as either large or small-volume based on the number of massive transfusion patients enrolled during the study period. We focused on estimation of parameters inspired by causal inference, specifically estimated impacts on patient outcomes related to the volume of the trauma hospital that treated them. We defined this association as the change in mean outcomes of interest that would be observed if, contrary to fact, subjects from large-volume sites were treated at small-volume sites (the effect of treatment among the treated). We estimated this parameter using three different methods, some of which use data-adaptive machine learning tools to derive the outcome models, minimizing residual confounding by reducing model misspecification. Differences between unadjusted and adjusted estimators sometimes differed dramatically, demonstrating the need to account for differences in patient characteristics in clinic comparisons. In addition, the estimators based on robust adjustment methods showed potential impacts of hospital volume. For instance, we estimated a survival benefit for patients who were treated at large-volume sites, which was not apparent in simpler, unadjusted comparisons. By removing arbitrary modeling decisions from the estimation process and concentrating on parameters that have more direct policy implications, these potentially automated approaches allow methodological standardization across similar comparativeness effectiveness studies.

## Introduction

Trauma is a leading cause of death worldwide and is an increasing global health burden, rapidly eclipsing HIV/AIDS, vaso-occlusive and infectious diseases [1,2], and understanding the underlying mechanisms and improving the treatment of trauma is of paramount clinical and public health interest. While there is a vast literature exploring the predictors and effects of the care of injured patients, this research is undertaken on an extremely heterogeneous group of patients in often austere and chaotic environments including the pre-hospital setting, the battlefield, the emergency department and the operating room. Confounding is introduced by the considerable heterogeneity among patients across trauma units, including differences in age, gender, injury types and severity. Finally, impacts of treatment decisions are often highly confounded by current patient health states. Indeed the very measures aimed at saving lives and improving outcomes often prevent clean separation of patient cohorts and make causal relationships challenging and sometimes impossible to discern. Missing data is also very prevalent because of the primacy of patient treatment over collecting data. Randomized controlled trials (RCTs), which minimize between-group differences and isolate a variable or treatment to be studied, are optimum, but are rare in trauma due to issues surrounding methodological complexity, issues with informed consent and expense.

There is a vast literature examining the impact of how and where care is delivered on patient outcomes, including treatment biases (e.g., large volume vs. small volume, urban vs. rural, American College of Surgeons verified, massive transfusion protocol vs. no massive transfusion protocol) [3–6]. Addressing the relative importance of center-level factors in the treatment of trauma is a challenging question, since each center has unique physicians, resources, and patient types. For example, it would not be fair to compare two centers with different distributions of baseline injury severity or other confounders. The ideal experiment would consist of one that could ignore geography and randomize the trauma centers such that an injured patient would have an equal likelihood of being treated at any center. The average outcomes across centers could then be compared. Instead, one must resort to statistically estimating such an association using available observational data. The bridge between the optimal experiment and the one available must involve dense data on patient characteristics and statistical methodologies that can *robustly* account for confounding differences of patient populations at different clinics.

Our approach described here utilizes two major advances in statistical methodology: (1) *targeted* data adaptive machine learning tools for modeling clinical outcomes and the distribution of patients across different trauma centers, given a potentially large number of covariates, and (2) using the resulting models to estimate parameters that reflect how the site characteristic of interest affects the distribution of outcomes among the patients. The purpose of this analysis was to present statistical methodology that can be ultimately available as an automated, data-adaptive statistical learner targeted towards the estimation of the impact of center-level characteristics on patient outcomes; we demonstrate the potential of these approaches on data from the PRospective, Observational, Multi-center Major Trauma Transfusion (PROMMTT) study [7].

## Methods

### Data: The PRospective, Observational, Multi-center Major Trauma Transfusion study

The PRospective, Observational Multi-center Major Trauma Transfusion (PROMMTT) study enrolled 1,245 individuals at ten level I trauma centers from around the United States [8]. Patients had to survive at least 30 minutes and receive at least one unit of red blood cells within 6 hours of arrival in the emergency department (ED) [8]. Once enrolled they were followed for diagnostic and therapeutic procedures and subsequent outcome data were collected [8]. The PROMMTT study was approved at each study site and the Data Coordinating Center by the local institutional review boards. The US Army Human Research Protections Office also provided secondary level review and approval. Patient records were de-identified prior to their use in this analysis.

For the purposes of this analysis, we partitioned centers and defined ‘volume’ based on the number of patients massively transfused, or receiving ten or more units of red blood cells in the first 24 hours, at each site. The top three massive transfusers were defined as large-volume centers while the others were defined as small-volume centers. We note that the hospitals could have been dichotomized by volume in many ways, and so the choice based upon volume of MT is somewhat arbitrary, and used for illustrative purposes only. Volume varies from a low of 4 patients in one hospital during the study period, to a high of around 80 patients, with no obvious inflection in MT volume between “large” and “small” groups. Thus, for the remainder of the paper, “large” versus “small” only refers to relative number of MT patients in this arbitrary grouping, with the split point placed between 32 and 33 MT patients, noting the analysis could be equivalently done for other categorizations, which, in turn, could have led to different estimates.

Only 1,242 of 1,245 PROMMTT patients had sufficient data to analyze and were used for the analysis. Table 1 shows the distributions of the outcomes of interest and other cohort characteristics in our sample. The three large-volume sites treated 551 individuals while 691 were treated at the seven small-volume sites.

**Table 1:** Summary statistics. Summaries are presented as percent where indicated, and mean (standard deviation) otherwise.

	Small Clinics (n = 691)	Large Clinics (n = 551)	*p* ^ for difference
**Explanatory Variables**			
Male (%)	73.7	75.1	0.555
Age (years)	42.2 (18.8)	39.0 (18.2)	***0*.*002*** *
Missing (%)	0	0.18	0.263
Ethnicity (%):			
Hispanic/Latino	18.8	19.8	0.667
Non-Hispanic/Latino	75.8	74.2	0.516
Missing	5.35	5.99	0.630
Race (%):			
White	74.8	58.1	***<0*.*001*** *
Black	15.6	20.9	***0*.*017*** *
Asian/Pacific Islander	3.62	3.99	0.731
Other	3.04	13.25	***<0*.*001*** *
Missing	2.89	3.81	0.369
BMI (kg/m^2^)	28.3 (7.16)	27.4 (6.32)	***0*.*028*** *
Missing (%)	13.6	32.8	***<0*.*001*** *
ISS	25.9 (15.8)	26.4 (14.5)	0.318
Penetrating injury (%)	31.4	40.1	***0*.*001*** *
Anticoagulant use (%):			
Yes	15.1	7.26	***<0*.*001*** *
No	59.6	72.8	***<0*.*001*** *
Missing	25.3	20.0	***0*.*026*** *
ED Systolic BP (mmHg)	110 (30.4)	106 (32.7)	***0*.*009*** *
Missing (%)	2.32	2.72	0.648
ED Heart rate (BPM)	106 (27.9)	106 (28.3)	0.761
Missing (%)	2.46	1.63	0.312
ED Glasgow coma score	9.51 (5.62)	10.1 (5.48)	0.195
Missing (%)	9.41	7.99	0.379
ED INR	1.40 (0.776)	1.52 (1.49)	0.064
Missing (%)	15.6	9.98	***0*.*003*** *
Partial thromboplastin time (s)	32.1 (18.7)	31.6 (16.2)	0.087
Missing (%)	17.9	13.6	***0*.*039*** *
ED Platelet count (10^9^/L)	223 (82.5)	243 (82.3)	***<0*.*001*** *
Missing (%)	4.92	6.35	0.274
ED Hemoglobin count (g/dL)	11.3 (2.37)	12.1 (2.23)	***<0*.*001*** *
Missing (%)	2.46	5.26	***0*.*009*** *
ED Base deficit (mEq/L)	-7.33 (5.69)	-6.70 (5.52)	0.075
Missing (%)	27.4	17.2	***<0*.*001*** *
**Outcomes**			
Mortality (%):			
2-hour	4.49	3.09	0.203
6-hour	8.39	7.99	0.795
24-hour	12.7	10.9	0.318
Overall	21.7	21.1	0.780
Complications (%)	6.66	3.63	***0*.*018*** *
Multiple organ failure (%)	1.59	1.09	0.449
Substantial bleeding (%):			
Yes	29.5	30.9	0.612
No	68.2	66.4	0.516
Unknown/Missing	2.32	2.72	0.648
Plasma infused by 24 hr (U)	4.77 (8.57)	7.92 (9.80)	***<0*.*001*** *
Platelets infused by 24 hr (U)	3.77 (7.68)	4.07 (8.34)	0.886
RBC infused by 24 hr (U)	7.91 (10.9)	8.59 (10.5)	***0*.*025*** *
Platelet:RBC ratio by 24 hr	0.372 (0.934)	0.343 (0.86)	0.805
Missing (%)	0.145	0.181	0.872
Plasma:RBC ratio by 24 hr	0.475 (0.634)	0.946 (0.731)	***<0*.*001*** *
Missing (%)	0.145	0.181	0.872

^**^**^ **p**-values derived from Mann-Whitney U and Z-tests for continuous and binary variables respectively.

* **p**-value significant (α = 0.05).

BMI: body mass index; ISS: injury severity score; BP: blood pressure; BPM: beats per minute; INR: international normalized ratio; RBC: red blood cell; U: units.

### Model and Parameter of Interest

The goal was to estimate the relationship between patient outcomes and site volume type, adjusting for a large number of covariates. If we define *Y* as the outcome, *S* (large vs. small-volume site) as the explanatory variable of interest, and *C* as all confounding variables, we could predict, on average, what would happen to a patient if they went to a site of a specific size, if we knew the model: *mean(Y | S = s*, *C)*. We discuss below how to estimate this prediction function, going beyond traditional regression approaches and utilizes machine-learning tools. First, however, we need to define a parameter that is not just a prediction for a specific patient (defined by their covariates *S* and *C*), but an association that summarizes the impact for each patient across the patient population. For instance, we could define the mean of these predictions, *mean(Y(s)) = mean{mean(Y | S = s*, *C)}*, where the outer mean is taken over all the patients (and the “natural” distribution of confounders). If the outcome prediction model is correct, along with important identifiability assumptions outlined below, we could then compare patient outcomes if, keeping their covariates (*C)* the same, they went to all large vs. small volume sites. This parameter, *mean(Y(*large*))−mean(Y(*small*))*, would be a measure of the average causal association of site size. We chose a slightly different parameter, which examines this difference only among the people who went to larger sites, or *mean{Y(*large*)−Y(*small*) | S =* large*}*. In what sub-group one estimates the causal association is somewhat arbitrary, but in this case we chose such a parameter because 1) these populations are often quite different, and a parameter over the whole study population has little meaning in some theoretical mixed target population of large and small hospitals and 2) we wish to have an effect estimate for which one could compare to a matching procedure discussed below, in which patients at large sites are paired to patients at small sites based on their characteristics. Our estimand of interest (based on explicit identifiability assumptions discussed below) is an effect of treatment among the treated (ETT):

ETT=mean{(mean[Y|S=large,C]−mean[Y|S=small,C])|S=large}. (1)

### Estimation: Regression Models

As mentioned above, we do not know the outcome prediction model *mean(Y | S = s*, *C)*, nor do we know how the distribution of center types differs by patient characteristics, *Prob(S =* large *| C)*, sometimes called a propensity score, and thus must estimate both functions from the data. While there are many options for the choice of prediction model to use, in order to make the most efficient use of available information and to estimate this prediction function using a theoretically optimal procedure, we advocate the use of the ensemble machine-learning algorithm called SuperLearner (SL). This prediction algorithm consists of a weighted combination of a library of prediction procedures [9]. We used 10-fold cross-validation where the data were divided randomly into 10 equal validation samples. One at a time, these validation samples were “removed” from the data, and prediction algorithms were fit on the remaining 9/10 of the data (the so-called training sample). Then, for each of these 10 overlapping training samples, the procedure predicted, for each algorithm, on the independent (independent of the corresponding training sample) validation sample. Finally, the SL procedure found the optimal weighted average of these predictors by comparing the observed outcomes to those predicted based on the models from the corresponding training sample. The procedure is more than an intuitive way to build a prediction model, as theory shows that the SL is guaranteed to perform as well as or better than the best prediction algorithm in the supplied library [9]. Thus, in essence one cannot beat SL, because any competing algorithm can be added as one of the candidates. Another of the great advantages of this approach is that it takes the art (and thus potential bias) out of model selection, as the user does not have to choose a particular prediction model but can allow all of them to be competitors, including very simple algorithms, very complicated machine learners, and even particular models that the researchers prefer. In this case, the SL builds the final model based on which of them does the best job of predicting a “new” set of data, so one need not engage in sterile arguments about the best approach to model such data—in this case “proof is in the pudding”.

Finally, though there was almost no missing values for the outcomes (see Table 1), some of the covariates have missing values for many of the observations. Instead of using an imputation approach, we create new basis functions: indicators of whether the variable is not missing (1 if not missing, 0 if missing, denoted by Δ) and the interaction of each indicator with its respective covariate (Δ*C*). The advantage of this approach is that this missingness information is used directly by the procedure to find the prediction model, so that if the missingness is informative, *and* there is sufficient information in the remaining measured variables to explain it relative to the outcome, then the procedure will find an optimal prediction model. Also, the necessary identifiability assumptions to get consistent estimates of causal effects under this strategy are very transparent—that is, the combination of observed covariates and missing data information must be sufficient such that there are no further unmeasured pathways related to the set of explanatory variables we include in the model.

In an observational clinical study like PROMMTT, some covariates of interest almost certainly remain unobserved due to life-saving measures trumping data collection. Since a patient’s condition could dictate whether or not information on that patient’s condition was recorded, missingness not at random (MNAR) cannot be ruled out here. Just as is the case with the assumption of no unmeasured confounding in observational studies, there is no single solution and in some circumstances one might conclude that the estimation is hopelessly biased due to the missing covariates, regardless of the missing data strategy employed. Here, our goal is to optimally estimate determinants of patient outcomes in the context of the available information without manufacturing additional information under potentially unsupported parametric assumptions. We therefore choose to employ the “indicator method” described above for any covariates with missing values, reported in Table 1.

### Estimation: Effect Estimates

We used several methods to estimate the ETT, the first being a simple substitution estimator [10], where one first predicts the outcome of interest based on predictors (*S* and *C*) to derive an estimate of *mean{Y | S*, *C}* and subsequently applies this model to predicting the outcome for subjects if one kept the *C* fixed, but changed the *S* to “large.” The average difference among these predicted values and the observed outcomes in large volume sites defines the estimate of the ETT. We did this approach both using an estimated main terms logistic regression model for *mean{Y | S*, *C}* (“Simple Subs.(regression)” in Table 2) as well as the same estimator based upon a SL fit using the *SuperLearner* [9,11] package in R [12]. For both, we derived the inference (the standard error) using the nonparametric bootstrap [13]. Though there is no theory that guarantees this estimator based upon SL has a normal limit distribution, we estimated the standard error using the nonparametric bootstrap and generated Wald-style confidence intervals (CI). For the estimator based upon main terms logistic regression, we can assume asymptotic normality but the CI is still not valid due to bias.

**Table 2:** Difference in means, and adjusted ETT estimates (simple substitution, TMLE, and propensity score matching). 95% confidence intervals are included in parentheses after each estimate. Asymptotic (normal-based) confidence intervals were calculated using the standard error of the Unadjusted, Targeted Maximum-Likelihood, and Matching estimators. The nonparametric bootstrap was used to generate 95% confidence intervals in the case of the Simple Substitution estimator.

		ETT			
Variable	Unadjusted Difference	Simple Subs. (regression)	Simple Subs. (SuperLearner)	TMLE	Matching
2-hour mortality	-0.014 (-0.035, 0.0071)	-0.017 (-0.041, 0.0084)	-0.018 (-0.039, 0.0091)	-0.016 (-0.039, 0.0078)	-0.029 (-0.071, 0.013)
6-hour mortality	-0.0041 (-0.035, 0.027)	-0.016 (-0.050, 0.019)	-0.031 (-0.071, 0.00097)	-0.030 (-0.075, 0.014)	***-0*.*10 (-0*.*17*, *-0*.*035)***
24-hour mortality	-0.018 (-0.054, 0.018)	-0.027 (-0.069, 0.018)	***-0*.*050 (-0*.*10*, *-0*.*010)***	***-0*.*061 (-0*.*11*, *-0*.*010)***	***-0*.*12 (-0*.*20*, *-0*.*046)***
Overall mortality	-0.0066 (-0.052, 0.039)	-0.00083 (-0.044, 0.045)	-0.0085 (-0.035, 0.054)	***-0*.*083 (-0*.*13*, *-0*.*033)***	***-0*.*17 (-0*.*26*, *-0*.*077)***
Complications	***-0*.*030 (-0*.*055*, *-0*.*0060)***	-0.019 (-0.046, 0.0078)	-0.022 (-0.045, 0.012)	-0.010 (-0.033, 0.012)	-0.011 (-0.051, 0.028)
Multiple organ failure	-0.0050 (-0.018, 0.0077)	0.0029 (-0.0081, 0.016)	0.0016 (-0.0056, 0.0095)	0.0045 (-0.0046, 0.014)	0.0047 (-0.015, 0.024)
Substantial bleeding	0.019 (-0.033, 0.071)	0.037 (-0.017, 0.093)	0.024 (-0.022, 0.079)	-0.043 (-0.12, 0.030)	-0.015 (-0.11, 0.081)
Plasma infused by 24 hr (U)	***3*.*1 (2*.*1*, *4*.*2)***	***3*.*8 (2*.*8*, *4*.*7)***	***3*.*3 (2*.*5*, *4*.*6)***	***2*.*2 (0*.*74*, *3*.*6)***	***2*.*1 (0*.*060*, *4*.*0)***
Platelets infused by 24 hr (U)	0.30 (-0.61, 1.2)	***1*.*2 (0*.*34*, *2*.*0)***	***0*.*79 (0*.*062*, *1*.*5)***	***1*.*1 (0*.*16*, *2*.*1)***	1.3 (-0.24, 2.8)
RBC infused by 24 hr (U)	0.68 (-0.52, 1.9)	***1*.*5 (0*.*40*, *2*.*8)***	***0*.*98 (0*.*27*, *2*.*0)***	-0.21 (-2.0, 1.5)	-0.46 (-2.8, 1.9)
Platelet:RBC ratio by 24 hr	-0.029 (-0.13, 0.071)	0.067 (-0.021, 0.15)	0.050 (-0.0056, 0.12)	0.058 (-0.043, 0.16)	0.11 (-0.034, 0.26)
Plasma:RBC ratio by 24 hr	***0*.*47 (0*.*39*, *0*.*55)***	***0*.*48 (0*.*40*, *0*.*55)***	***0*.*46 (0*.*39*, *0*.*53)***	***0*.*36 (0*.*26*, *0*.*47)***	***0*.*35 (0*.*21*, *0*.*49)***

This substitution estimator can be updated to achieve additional bias reduction via a so-called targeted maximum-likelihood estimation (TMLE) step. The targeting procedure updates the estimated conditional mean functions *mean{Y | S*, *C}* and *Prob(S =* large *| C)* by adding “clever covariates” to the original respective SL regression fits. In each case, a logistic regression of the outcome (*Y* or *S*) on its respective clever covariate is estimated, with the log-odds of the previously predicted outcome for each patient serving as an offset. When the procedure converges (meaning that the resulting logistic regression coefficients no longer change measurably between iterations), the resulting updated function *mean{Y | S*, *C}* can be evaluated at each level of the treatment to yield a targeted estimate of *mean{Y | S =* large, *C}−mean{Y | S =* small, *C}*, evaluated only among those treated at a large volume site. This estimator has additional desirable statistical properties that the original, non-targeted substitution estimator does not; the additional step can further reduce bias *and* results in normally distributed estimators, whereas the original substitution estimator based upon SL alone is not guaranteed to have a normal limit distribution (see chapter 8 in [14]).

Finally, the matching estimator uses the SuperLearner to estimate the propensity score, *Prob(S =* large *| C)*, and matches each large volume site patient to the closest small volume site patient based on this score (in this case, with replacement and allowing ties). Any small volume site patient whose propensity score was not within 0.05 standard deviations of any large volume site patient’s score was discarded for the purposes of this estimator. Here, the ETT is estimated by the average of the difference in the outcomes among these matched sites [15,16].

We note that the substitution estimator and the matching estimator rely on consistently estimating the prediction model and propensity score model, respectively. However, the TMLE relies on only estimating one of these consistently, and thus has greater robustness to model misspecification than either of these competitors.

## Results

We show the bivariate empirical distribution of covariates (*C*) by site in Table 1. Though this does not show the differences in the joint distribution of the explanatory variables, there appear to be important bivariate differences among them; the large hospitals serve a more racially diverse, younger group of patients who have more penetrating injuries, less baseline use of anticoagulants (probably related to the younger age), and higher platelet and hemoglobin counts observed in the emergency department (ED). There are also statistically significant unadjusted differences in the mean outcomes in large versus small-volume hospitals, particularly a lower proportion of complications, more infusion of platelets and RBCs, as well as a higher ratio of plasma to RBCs. These differences underscore of using estimators that can robustly incorporate differences in distribution of baseline characteristics in estimates of the marginal impact of site-level characteristic.

Table 2 contains the estimated unadjusted and adjusted (ETT by various methods) associations. Complications, for which the unadjusted estimator showed significantly lower rates in the larger-volume hospitals, did not significantly differ after adjustment using any of the ETT estimators. Conversely, overall mortality was significantly lower in larger hospitals after adjustment among the robust ETT estimators; the TMLE and matching estimators yielded 8.3% (95% CIs: (-13%, -3.3%)) and 17% (95% CIs: (-26%, -7.7%)) differences, respectively. We note that the matching estimator excludes patients in large volume sites if they cannot be matched with a patient in a small-volume site, and in this case 51 out of the 551 subjects at the large volume sites were found to be outside the p-score caliper of 0.05 standard deviations of any patient at the small sites. Thus, the actual parameter estimated is not quite the ETT, but only the effect among the subset of the population that can be matched. The advantage of this approach is that this estimator avoids extrapolating the impact of site size from groups defined by the covariates where there are insufficient numbers of large and small volume patients. The disadvantage is that the parameter is conditioned on a random subset, and thus its interpretation could be problematic.

Plasma infusion volume differs significantly, and adjustment combined with the use of robust estimators only modestly diminishes the estimated mean difference among those who were treated at a large volume center, implying less confounding by characteristics in Table 1. However, the volume of platelets infused is significantly different after adjustment using the TMLE estimator, and marginally significant (or close to significance) with the matching estimator. Thus, the robust semi-parametric (data-adaptive) estimators find important differences among these hospitals that are not always apparent in simpler comparisons. This is due to both confounding and also misspecification when arbitrary modeling assumptions (e.g., main terms logistic regression are used) are made.

### Diagnostics

One of the challenges of using flexible, machine-learning tools is how to examine the fit of such models, and also how to explore which variables have the greatest influence on the resulting estimators. Though having a simple regression model that one can examine relatively easily in more traditional approaches seems an advantage, it is generally illusory. Because a restricted parametric model is likely to be misspecified, the resulting coefficients, for instance, are likely to have unclear interpretations, so their attractiveness is more the appearance of simplicity than the actual utility of interpretation [17]. An estimator like the TMLE, on the other hand, allows for use of more flexible, nonparametric prediction models, is protected against misspecification of either the treatment mechanism or the conditional distribution of the outcome, and uses the information in the data in the most efficient manner for estimating a *target* parameter. One could consider as a downside of these techniques their reliance on “black-box” algorithms, which, without further exploration, are not easily interpreted. However, below, we provide some example tools for exploring the results reported in Table 2, which help to explain how this automated, data-adaptive procedure results in the final estimates.

### Effect of patient differences on overall mortality

**Fig 1:**
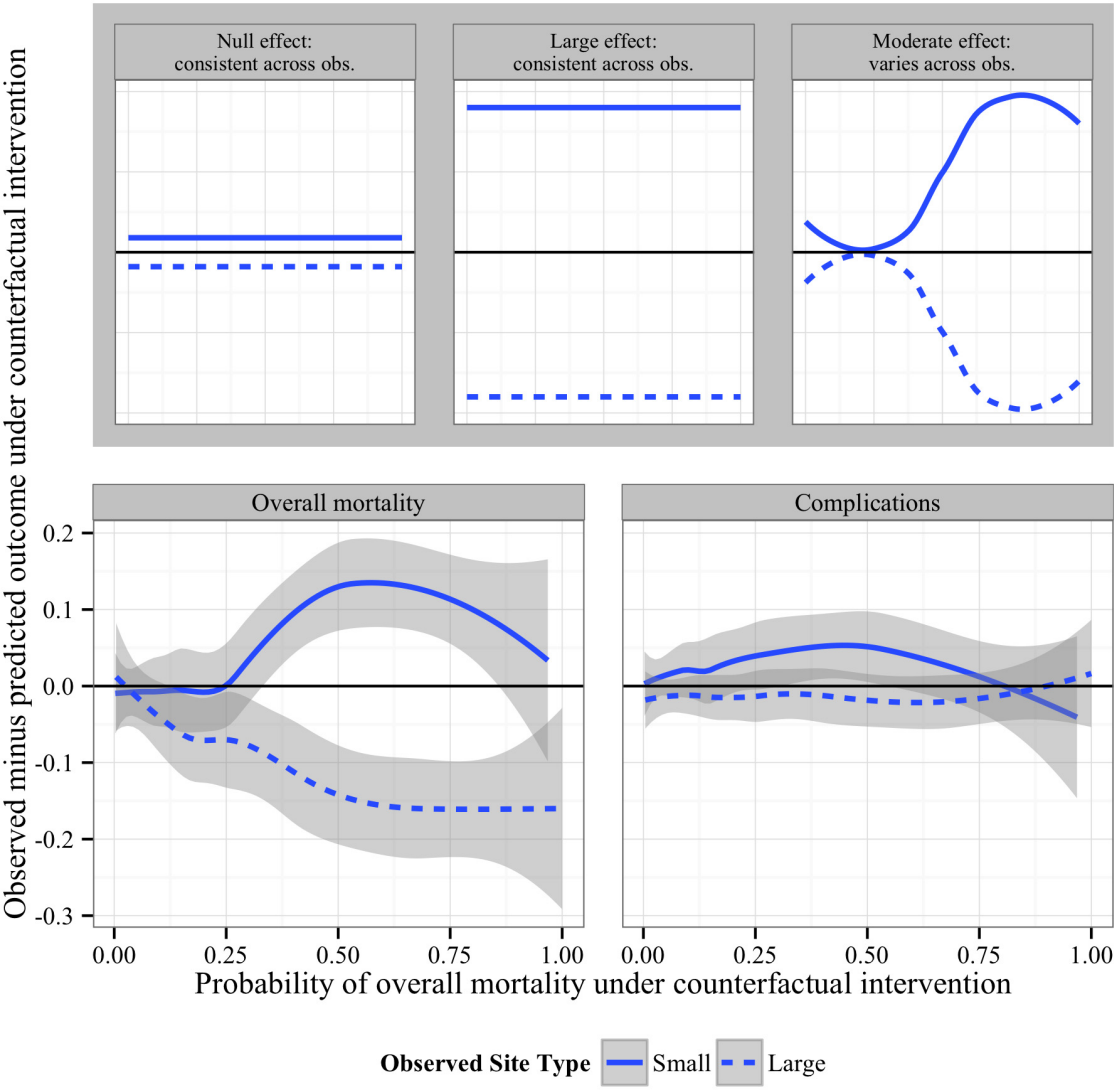
“Counterfactual” residual plot for binary outcomes. Residuals (see *Diagnostics*) for each outcome were plotted against probabilities of overall mortality for large (dotted line) and small-volume (solid line) site patients for binary outcomes using loess smooths.

We demonstrate these tools by examining the results for overall mortality. The goal is to identify which patients have the biggest impact on the estimates of the ETT, and perhaps are those most sensitive to any differences in practice at these clinics. To do so, we calculated “counterfactual” residuals for each observation among those in the *S =* large clinics: *Y—predicted(Y | S =* small, *C)*, or the difference of the observed outcome and that predicted according to the resulting model had the *C* stayed the same, but the clinic size switched from its observed value to “small.” Positive residuals identify those subjects who have an estimated increase in outcome possibly due to hospital-volume, while those with negative values display the opposite. One could group or cluster the patients in various ways to examine if there are some patients that appear to have been more affected by going to a small versus large-volume hospital. We did so by ordering the patients using their predicted probability of death based on our SL model; this results in a meaningful ranking of patients from those are lower to higher risk of the outcome based upon the constellation of clinical and demographic factors. Specifically, a loess smooth of these residuals versus simply their predicted outcome (in this case, *predicted(Y | S =* large, *C))* was examined. This allows one to estimate the difference in mortality due to volume versus patients on a scale from lower to higher estimated risk of death. We repeat the converse of this for subjects from smaller sites. The full results are presented in S1 and S2 Figs, for binary and continuous outcomes, respectively, while Fig 1 displays a subset of the results contained in S1 Fig.

The top inset of Fig 1 gives schematics for what these plots might look like under three scenarios: 1) there is no difference in the estimated ETT for the outcome (two horizontal lines close to the null line of 0), 2) there is a significant difference, but all patients are affected equally (two horizontal lines away from the null line), and 3) some patients (in this case, patients at a higher risk of death) are more affected by whether they end up at a large versus small volume hospital. For those outcomes where the estimate of ETT was close to the null, both the large and small volume hospital curves are near the null line at 0 (e.g., complications). For overall mortality, among those individuals in smaller hospitals, those with greater estimated probability of overall mortality appear to contribute the most to the estimate of greater increase in mortality among smaller sites, though this difference gets smaller for the most severe (highest probability of death) patients. Though it is not appropriate to reference the 95% confidence bands as reliable inference (given this is post-hoc), these exploratory plots provide a way to decompose the estimated differences seen in Table 2.

In order to characterize the individuals whose mortality would have been most affected by a site type change, we compared patients on either side of the point at which the loess curves started to diverge from 0, an area highlighted in blue in S4 Fig. This identified 148 individuals with negative residuals (survived) and 68 with positive residuals (died) who were treated at a large volume site and were estimated to be most affected by a theoretical change from a small to large volume site. These patients were then compared to the remaining large volume site patients in the white area of S4 Fig —those on whom the site volume appeared to have, on average, less of an impact. Patients who were estimated to be most affected by the site change were significantly more likely to be Black, had higher blunt injury rates, were more severely injured, and were further from consciousness (S1 Table). They also had significantly longer partial thromboplastin times, more severe base deficits, smaller INRs, and lower platelet counts (S1 Table). They were more likely to experience multiple organ failure and had higher transfusion rates, as well as lower rates of transfused red blood cells relative to other blood products (S2 Table).

To further illuminate which types of patients were driving the estimated difference due to hospital volume, we constructed a histogram of counterfactual predicted probabilities of mortality, by site (S3 Fig). Patients from all sites span the entire range of probabilities, suggesting that the curves fit to the residuals (Fig 1, S1 and S2 Figs) are not driven by only a few individuals.

### Propensity Score Matching Follow-up

**Fig 2:**
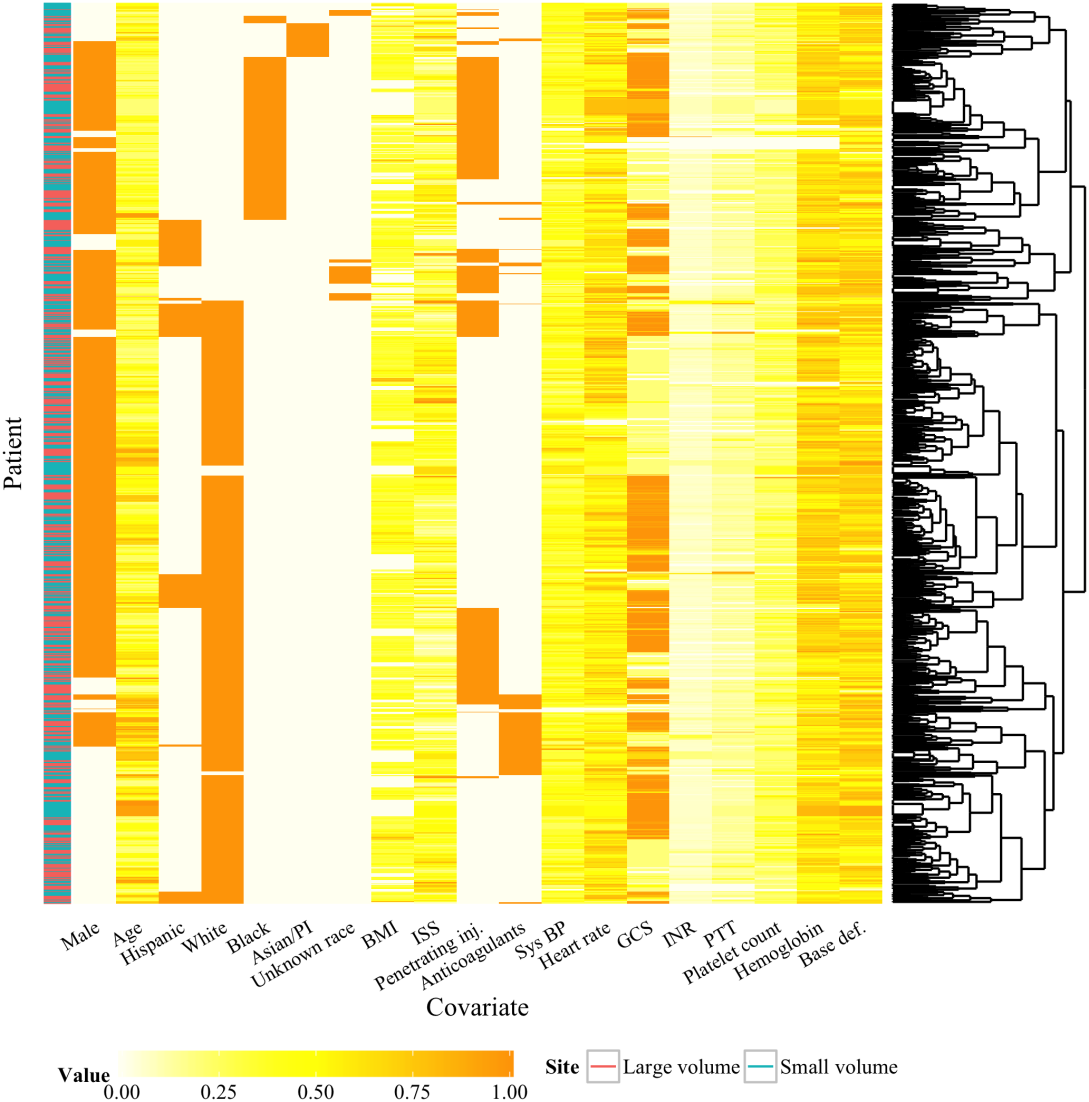
Heatmap of matched data set with hierarchical clustering. A visualization of the scaled covariate values in the matched data set with hierarchical clustering of the individual revealed little clustering of the individuals by site type, indicated in the bar on the left, suggesting that balance in the covariates was achieved. The dendrogram on the right is the result of hierarchical clustering of the individuals based on their covariate values. The color bar on the left indicates the site size where each individual was treated (purple corresponds to small-volume sites and blue to large-volume sites).

In addition to the supplementary analysis on the subset of patients whose mortality probabilities were estimated to be most impacted by a site type change, we explored the matched data set generated by the propensity score procedure to determine whether the procedure achieved balance in the covariates in the large versus small volume comparisons. The heatmap in Fig 2 is a visualization of the entire matched data set where the data have been scaled to be comparable across different variables. This procedure provides an exploration of the joint distribution of variables across individuals to see if balance was achieved across the multivariate distribution of adjustment variables. If the matching had not been successful, we would expect to see clusters of individuals corresponding to groupings based on the hospital volume (large versus small). While we see distinct clusters of individuals based on their covariate values, this appears not to coincide with being in a large versus small volume site, suggesting that reasonable balance in the covariates (via propensity score matching) occurred.

## Discussion

We here provide a general method for the objective comparison of the patient outcomes at different clinical sites using a parameter motivated by the causal inference literature and making use of potentially automatable, machine-learning techniques for data-adaptive estimation. Our data indicates that there are significant differences in outcomes based on the size of the site at which a patient was treated. Treated in this manner the data suggests a mortality benefit for those who were treated at centers that had the largest numbers of massively transfused patients. Interestingly we show here that this difference arose primarily in patients in the middle of the “prognostic” severity scale, that is, patients that neither had very low or very high estimated probability of death based on baseline factors. Further exploration within this moderately injured group of patients revealed both higher estimated probabilities of mortality and lower estimated probabilities of being massively transfused if they had been treated at small-volume sites (as opposed to large-volume sites), which supported the hypothesis that transfusion practices could be driving this observed mortality benefit. We subsequently found that the large-volume patients who would have been most affected by being treated at a small-volume site were indeed bleeding more and were generally worse off than their comparison group, which could indicate a need for more aggressive and balanced transfusion practices.

Uncontrolled bleeding remains the single largest contributor to preventable 24-hour mortality, making the rapid identification and treatment of hemorrhage after trauma critically important [18]. Hence it is plausible that the centers with larger volumes more judiciously identified those who were in need of early initiation of massive transfusion, which has been shown in prior studies to improve mortality in patients with potentially survivable injuries [19–21]. Large volume centers may have been able to respond earlier with a balanced transfusion, conferring an additional advantage. In contrast, those with nearly fatal or universally fatal injury were expected to die no matter what treatment they received.

Upon further investigation (presented in S1 Table), the patients who were less affected by the site switch generally had higher Glasgow coma scores, lower injury severity scores, higher rates of penetrating injury, were less acidotic and less coagulopathic (evidenced by, on average, shorter partial thromboplastin times and higher platelet counts). They also had lower probabilities of some negative outcomes and generally required less transfusion of blood products (displayed in S2 Table). These data support the findings that those who are mildly injured are far less likely to be suffering from catastrophic hemorrhage and will be more likely do well, regardless of the center. In any case, the results suggest a potential difference in average care in the two groups of sites, which would motivate more finely collected data to highlight either heterogeneity in treatment that should be addressed, or differences in patient groups unaccounted for in the variables in our analysis.

By estimating the ETT and adjusting for differences in patients across sites as aggressively as possible, we were able to examine differences in the distribution of outcomes for patients at large-volume trauma centers that might be related to heterogeneity in care across sites. This process highlights the utility of the combination of machine learning and causal inference modeling in clinical research and allows for a comparison that would have been infeasible in a randomized experiment. The estimation of this comparison could be carried out using several methods. We utilized machine learning approaches in both propensity score matching estimates and those based on the outcome regression models. These approaches are advantageous because they allow us to avoid relying on unnecessary modeling assumptions. For the majority of the outcomes examined here, the direction of estimated effects from each of the four ETT approaches was the same and the magnitudes were comparable, suggesting that each approach was capturing some underlying differences between the centers. The unadjusted comparisons demonstrate how a naïve approach to comparing the centers that does not adjust for patient characteristics could miss some important outcomes that did in fact differ across the sites. Propensity score matching is another intuitive approach that can be used to generate counterfactual outcomes for individuals by matching them to patients from other sites, and we showed that balance was achieved in the covariates with a multivariate analysis of the matched data set. These more commonly used approaches provided similar results to the TMLE, which is an estimator designed to achieve the optimal bias-variance tradeoff and that has other desirable statistical properties [14,22].

Such rigorous comparisons of center-level factors have the potential to be a useful tool for identifying areas of improvement for individual centers and standardizing care across centers. This is where further investigation can yield improvements in patient outcomes. In this case, we partitioned the PROMMTT centers based on the number of patients they massively transfused. All of the centers in PROMMTT are acknowledged to be leading high-quality level I trauma centers and hence we understand that a limitation to this analysis is that the cutoff of ‘large volume’ vs. ‘small volume’ centers is somewhat arbitrary. Other partitions are possible, and this general methodology would apply regardless of the choice. Ultimately, our results represent a proof of concept that that this approach can be used for any comparisons of hospital care characteristics that are of clinical interest in the context of high-dimensional patient and clinical measures.

The estimators implemented in this paper are more complex than other approaches, but permit a less biased, machine-learning approach to estimation of both prognostic models and resulting estimates of potential interventions. As in most observational clinical studies, it is likely that we did not include all confounders of the effect volume on the outcomes of interest. However, these data-adaptive approaches are so attractive because they optimize model/variable selection in the context of high-dimensional patient information. This is by no means the only approach to comparing patient outcomes at different hospitals. Indeed, additional comparisons could examine more closely what factors in particular are responsible for the differences in site effectiveness. Given the proliferation of interest in comparative effectiveness, familiarity with these data-adaptive (machine learning) causal inference methods will allow for a better understanding of factors influencing trauma patient care and provide directions towards other areas for improvement.

## Supporting Information

S1 Table Means and standard deviations of covariates in the subsets defined by the magnitude of residuals. Groups (large versus small) are defined in S4 Fig (in versus out of blue area). P-value included for comparison (based on t-test or Pearson’s chi-square test for ordered versus binary outcomes, respectively).
(DOCX)

S2 Table Means and standard deviations of outcomes in the subsets defined by the magnitude of residuals. Groups (large versus small) are defined in S4 Fig (in versus out of blue area). P-value included for comparison (based on t-test or Pearson’s chi-square test for ordered versus binary outcomes, respectively).
(DOCX)

S1 Information Further explanation regarding supplementary tables and figures. (DOCX)

S1 Fig “Counterfactual” residual plot for binary outcomes. Residuals (see *Diagnostics*) for each outcome were plotted against probabilities of overall mortality for large (dotted line) and small-volume (solid line) site patients for binary outcomes using loess smooths.
(TIF)

S2 Fig Counterfactual residual plot for continuous outcomes. Counterfactual residuals for each outcome plotted against probabilities of overall mortality for large (dotted line) and small-volume (solid line) site patients for continuous outcomes.
(TIF)

S3 Fig Histogram of counterfactual predicted probabilities of overall mortality. Histogram of the number of individuals supporting the loess curves in the residual plots in S1 and S2 Figs colored by site membership. While there are more individuals at lower probabilities, there are individuals from various sites across the entire range of probabilities suggesting that the loess curves in the residual plots are not driven by only a few individuals.
(TIF)

S4 Fig Patients driving the residuals for overall mortality. The blue area indicates where the loess curves deviated from zero. We compared individuals with positive and negative residuals in the large-volume subset.
(TIF)
