# Supplementary material for: Statistical Machines for Trauma Hospital Outcomes Research: Application to the PRospective, Observational, Multi-Center Major Trauma Transfusion (PROMMTT) Study
Source: PLoS One. 2015 Aug 21;10(8):e0136438. doi: 10.1371/journal.pone.0136438 (PMC4546674; doi:10.1371/journal.pone.0136438)
Supplement: S1 Table — Groups (large versus small) are defined in S4 Fig (in versus out of blue area). P-value included for comparison (based on t-test or Pearson’s chi-square test for ordered versus binary outcomes, respectively). (DOCX) [file pone.0136438.s001.docx]

|  | **Mean among those *least* affected by site volume** | **Mean among those *most* affected by site volume** | **P-value** |
| --- | --- | --- | --- |
| **Male** | 0.739 (0.44) | 0.768 (0.423) | 0.434 |
| **Age (years)** | 36.7 (16.7) | 41.9 (19.7) | ***0.005*** |
| **Hispanic ethnicity** | 0.232 (0.423) | 0.152 (0.36) | ***0.019*** |
| **Race: White** | 0.58 (0.494) | 0.582 (0.494) | 0.95 |
| **Race: Black** | 0.201 (0.401) | 0.219 (0.415) | 0.591 |
| **Race: Asian/Pacific Islander** | 0.0414 (0.2) | 0.038 (0.192) | 0.839 |
| **Race: Unknown** | 0.0159 (0.125) | 0.038 (0.192) | 0.103 |
| **BMI (kg/m^2^)** | 27.2 (5.98) | 27.7 (6.84) | 0.573 |
| **ISS** | 20.9 (12.4) | 33.6 (14.1) | ***<0.001*** |
| **Penetrating injury** | 0.484 (0.501) | 0.291 (0.455) | ***<0.001*** |
| **Anticoagulant use** | 0.0854 (0.28) | 0.1 (0.301) | 0.608 |
| **ED Systolic BP (mmHg)** | 105 (28.8) | 106 (37.4) | 0.938 |
| **ED Heart rate (BPM)** | 105 (25.9) | 108 (31.2) | 0.218 |
| **ED Glasgow coma score** | 12 (4.78) | 7.29 (5.19) | ***<0.001*** |
| **ED INR** | 1.39 (1.35) | 1.71 (1.67) | ***<0.001*** |
| **Partial thromboplastin time (s)** | 28.3 (10.9) | 37 (21.3) | ***<0.001*** |
| **ED Platelet count (10^9^/L)** | 254 (76.1) | 229 (88.4) | ***<0.001*** |
| **ED Hemoglobin count (g/dL)** | 12.2 (2.16) | 12 (2.33) | 0.799 |
| **ED Base deficit (mEq/L)** | -5.47 (4.76) | -8.34 (6.04) | ***<0.001*** |
