# Supplementary material for: Statistical Machines for Trauma Hospital Outcomes Research: Application to the PRospective, Observational, Multi-Center Major Trauma Transfusion (PROMMTT) Study
Source: PLoS One. 2015 Aug 21;10(8):e0136438. doi: 10.1371/journal.pone.0136438 (PMC4546674; doi:10.1371/journal.pone.0136438)
Supplement: S2 Table — Groups (large versus small) are defined in S4 Fig (in versus out of blue area). P-value included for comparison (based on t-test or Pearson’s chi-square test for ordered versus binary outcomes, respectively). (DOCX) [file pone.0136438.s002.docx]

|  | **Mean among those *least* affected by site volume** | **Mean among those *most* affected by site volume** | **P-value** |
| --- | --- | --- | --- |
| **2-hour mortality** | 0.0159 (0.125) | 0.0506 (0.22) | ***0.02*** |
| **6-hour mortality** | 0.0637 (0.245) | 0.101 (0.302) | 0.107 |
| **24-hour mortality** | 0.0732 (0.261) | 0.156 (0.364) | ***0.002*** |
| **Complications** | 0.0255 (0.158) | 0.0506 (0.22) | 0.118 |
| **Multiple organ failure** | 0.00318 (0.0564) | 0.0211 (0.144) | ***0.045*** |
| **Substantial bleeding** | 0.261 (0.44) | 0.422 (0.495) | ***<0.001*** |
| **Plasma infused by 24 hr (U)** | 5.73 (7.49) | 10.8 (11.6) | ***<0.001*** |
| **Platelets infused by 24 hr (U)** | 2.71 (6.8) | 5.87 (9.74) | ***<0.001*** |
| **RBC infused by 24 hr (U)** | 6.95 (8.49) | 10.8 (12.3) | ***<0.001*** |
| **Platelet:RBC ratio by 24 hr** | 0.22 (0.529) | 0.504 (1.14) | ***<0.001*** |
| **Plasma:RBC ratio by 24 hr** | 0.811 (0.661) | 1.12 (0.779) | ***<0.001*** |
