# Supplementary material for: Statistical Machines for Trauma Hospital Outcomes Research: Application to the PRospective, Observational, Multi-Center Major Trauma Transfusion (PROMMTT) Study
Source: PLoS One. 2015 Aug 21;10(8):e0136438. doi: 10.1371/journal.pone.0136438 (PMC4546674; doi:10.1371/journal.pone.0136438)
Supplement: S1 Information — (DOCX) [file pone.0136438.s003.docx]

# Supporting Information

In order to characterize the individuals whose mortality would have been most affected by a site type change, we compared patients on either side of the point at which the loess curves started to diverge from 0, an area highlighted in blue in Figure S4. This identified 146 individuals with negative residuals (survived) and 52 individuals with positive residuals (died) who are estimated to be most affected by a theoretical change from small to large volume sites – these are then compared to the remaining subjects in the “white” area of the plot – those that appear to have, on average, less of an impact due to site. These two groups are compared in Table S1, and patients who were estimated to be affected by the site change were older, had higher blunt injury rates, larger injury severity scores (ISS), and higher heart rates. They also had significantly longer partial thromboplastin times, more severe base deficits, higher INRs, and were further from consciousness (evidenced by lower Glasgow coma scores). They were more likely to experience bleeding events and had higher transfusion rates (Table S2).
