# Supplementary figures and images for: Statistical Machines for Trauma Hospital Outcomes Research: Application to the PRospective, Observational, Multi-Center Major Trauma Transfusion (PROMMTT) Study
Source: PLoS One. 2015 Aug 21;10(8):e0136438. doi: 10.1371/journal.pone.0136438 (PMC4546674; doi:10.1371/journal.pone.0136438)

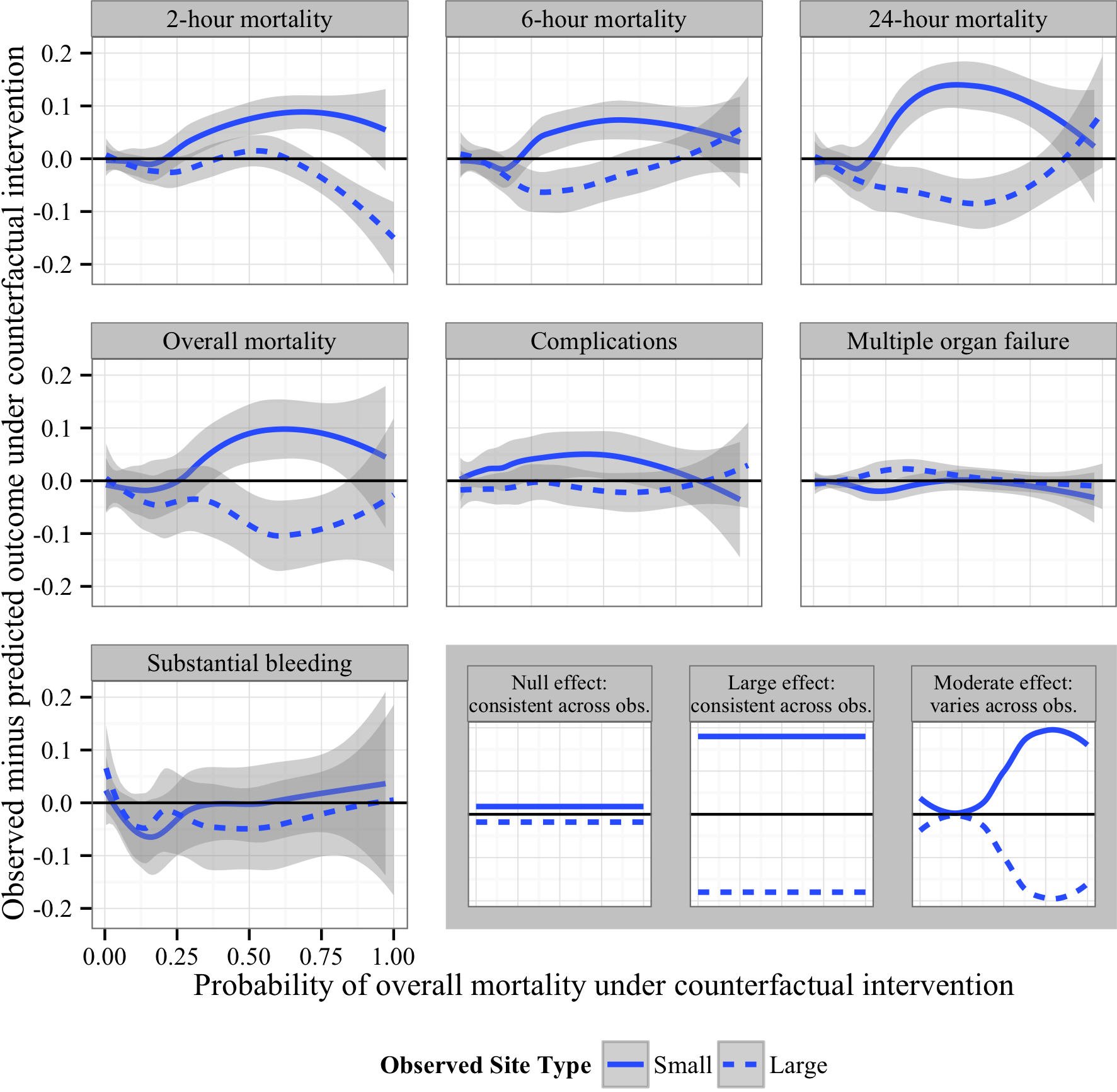

Supplement: S1 Fig — Residuals (see Diagnostics) for each outcome were plotted against probabilities of overall mortality for large (dotted line) and small-volume (solid line) site patients for binary outcomes using loess smooths. (TIF) [file pone.0136438.s004.tif]

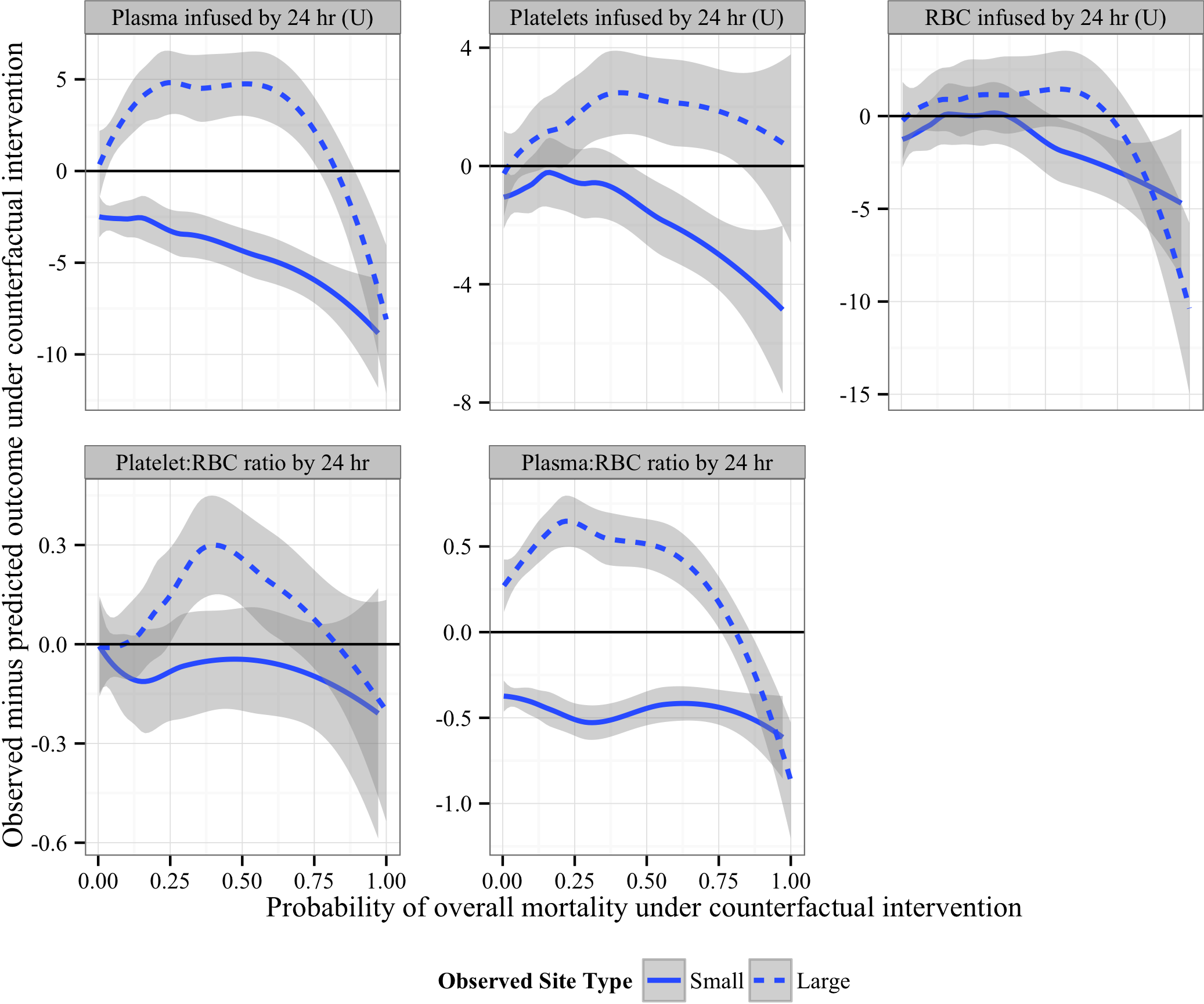

Supplement: S2 Fig — Counterfactual residuals for each outcome plotted against probabilities of overall mortality for large (dotted line) and small-volume (solid line) site patients for continuous outcomes. (TIF) [file pone.0136438.s005.tif]

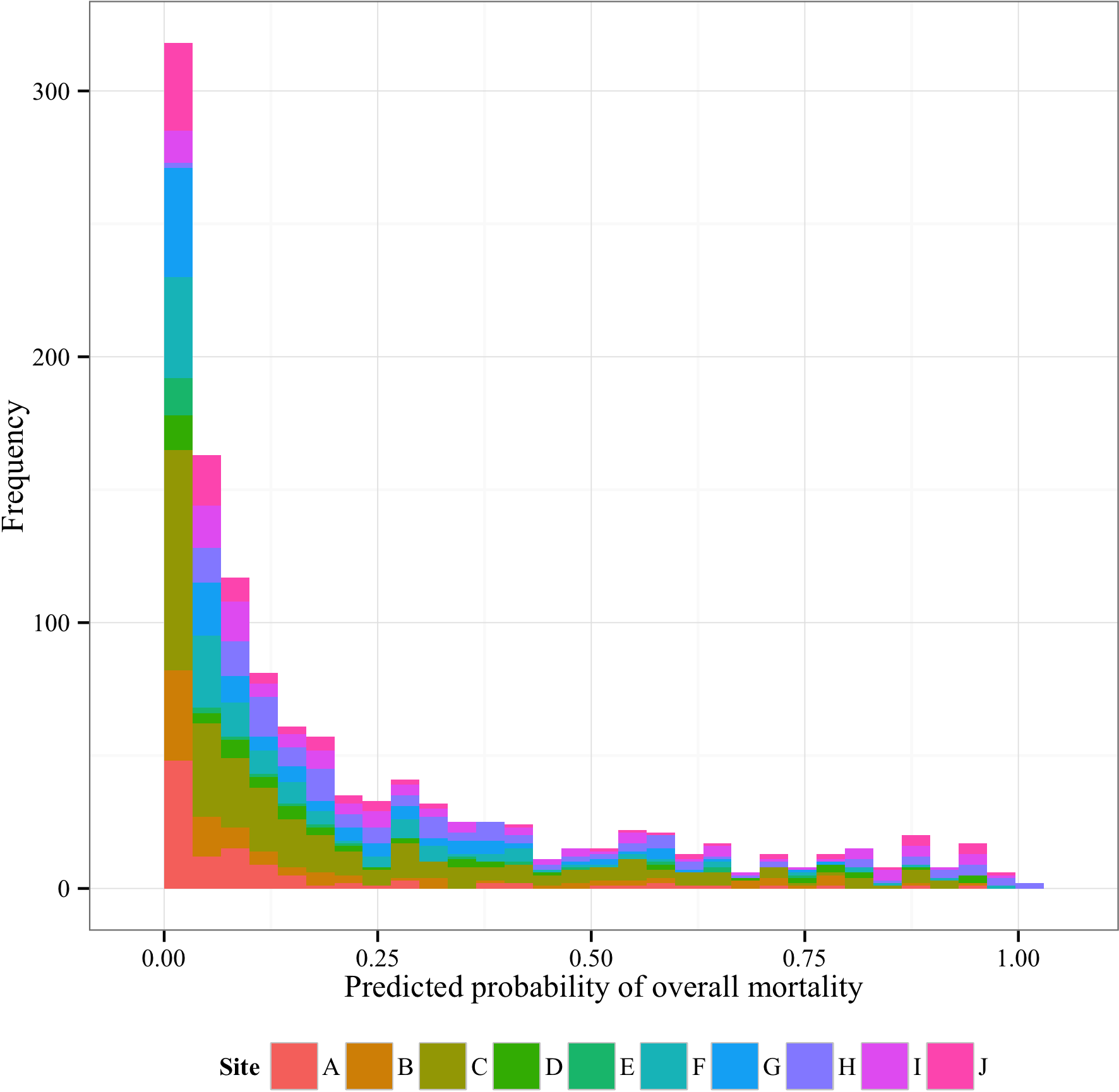

Supplement: S3 Fig — Histogram of the number of individuals supporting the loess curves in the residual plots in S1 and S2 Figs colored by site membership. While there are more individuals at lower probabilities, there are individuals from various sites across the entire range of probabilities suggesting that the loess curves in the residual plots are not driven by only a few individuals. (TIF) [file pone.0136438.s006.tif]

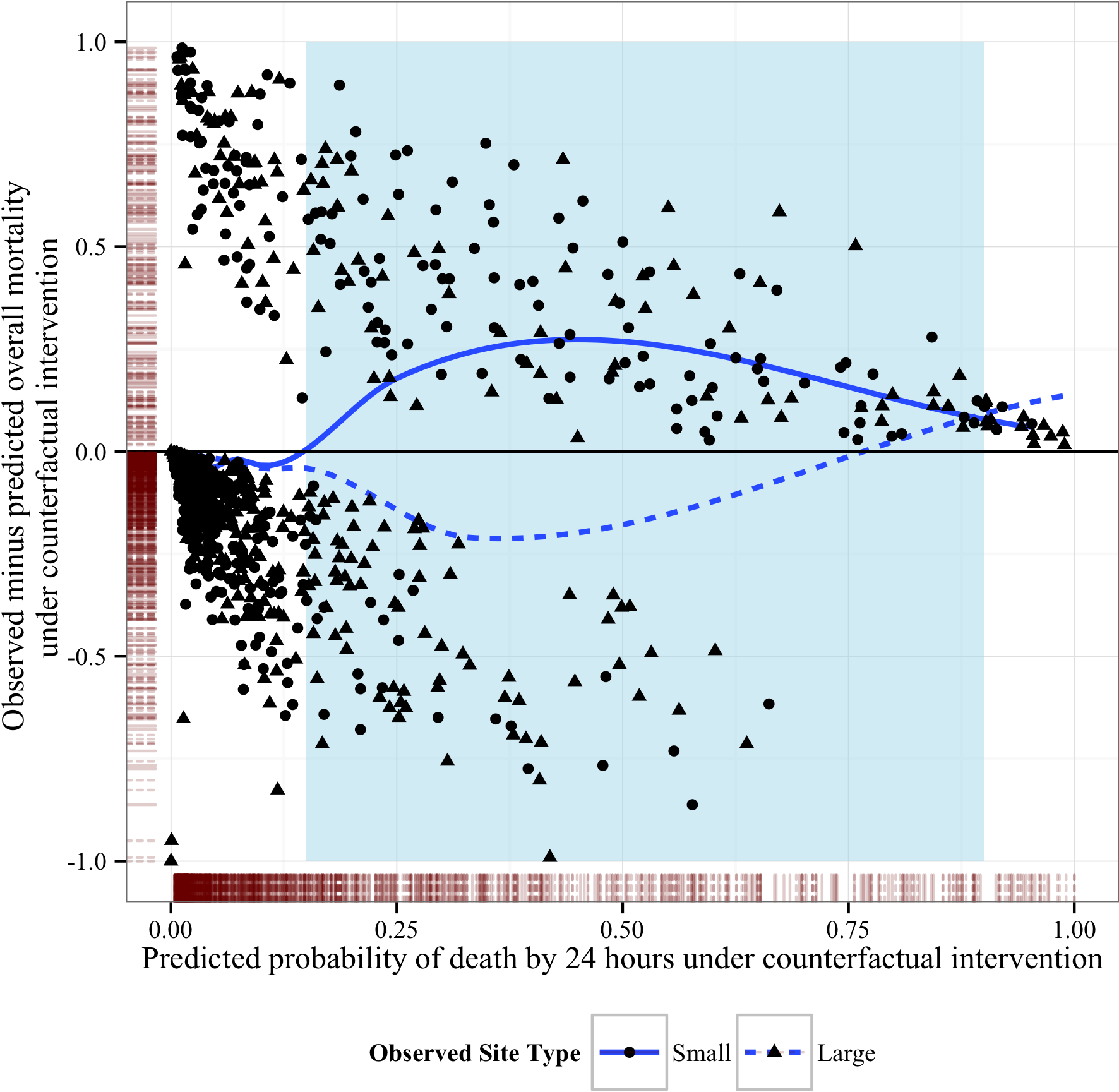

Supplement: S4 Fig — The blue area indicates where the loess curves deviated from zero. We compared individuals with positive and negative residuals in the large-volume subset. (TIF) [file pone.0136438.s007.tif]
